# Gas Phase Synthesis of Multi-Element Nanoparticles

**DOI:** 10.3390/nano11112803

**Published:** 2021-10-22

**Authors:** Raúl López-Martín, Benito Santos Burgos, Peter S. Normile, José A. De Toro, Chris Binns

**Affiliations:** Departamento de Física Aplicada, Instituto Regional de Investigación Científica Aplicada (IRICA), Universidad de Castilla la Mancha, 13071 Ciudad Real, Spain; raul.lopez@uclm.es (R.L.-M.); benito.santos@uclm.es (B.S.B.); peter.normile@uclm.es (P.S.N.); joseangel.toro@uclm.es (J.A.D.T.)

**Keywords:** nanoparticle, Janus, core–shell, alloy

## Abstract

The advantages of gas-phase synthesis of nanoparticles in terms of size control and flexibility in choice of materials is well known. There is increasing interest in synthesizing multi-element nanoparticles in order to optimize their performance in specific applications, and here, the flexibility of material choice is a key advantage. Mixtures of almost any solid materials can be manufactured and in the case of core–shell particles, there is independent control over core size and shell thickness. This review presents different methods of producing multi-element nanoparticles, including the use of multiple targets, alloy targets and in-line deposition methods to coat pre-formed cores. It also discusses the factors that produce alloy, core–shell or Janus morphologies and what is possible or not to synthesize. Some applications of multi-element nanoparticles in medicine will be described.

## 1. Introduction

It is well known that sufficiently small particles, with diameters at the nanometer scale (nanoparticles or NPs), have properties that deviate from the bulk material; in addition, their properties become dependent on their size, which is in itself novel [1]. These attributes arise from the high proportion of under-coordinated surface atoms [2] and from quantum size effects, both of which vary with cluster dimension. With the advent of methods capable of synthesizing nanoparticles with precisely controlled size [3], including, in some cases, with a specified number of atoms in a particle [4], many interesting discoveries have been made. These include magic numbers of atoms that produce enhanced stability of nanoparticles [4], novel atomic and electronic configurations [5] and the appearance of special magnetic [6,7,8,9,10] and optical [11,12] properties. If one considers a nanoparticle as a building block of a bulk material, then, since it is possible to tailor the fundamental properties of the building block just by changing its size, it is as if a third dimension is added to the periodic table. Thus, for each element, changing the size of the constituent nanoparticles modifies the properties of the material produced.

Synthesis of NPs with controlled size, shape and composition is therefore a “hot topic” in materials science. The methods used can be broadly classified as wet chemistry [13,14,15], exploitation of biological processes [16,17], mechanical milling [18] and gas-phase synthesis. In this review, the focus lies on gas-phase methods, which offer the most precise size control and the greatest flexibility of choice of elements, especially with the production of multi-element NPs. In addition, NPs can be produced without the presence of ligands or surfactants, which can change their properties [19,20,21], though these can be added later if required. A further advantage, especially with ultra-high vacuum- (UHV-) based sources, is that nanoparticles can be prepared free of oxides, or they can be oxidized with a high degree of control. Traditionally, the main drawback of the technique has been its relatively low yield [3], but more recently, certain designs have led to significantly higher deposition rates [3]. The various gas-phase methods for synthesizing NPs are described in the following section.

**Figure 1:**
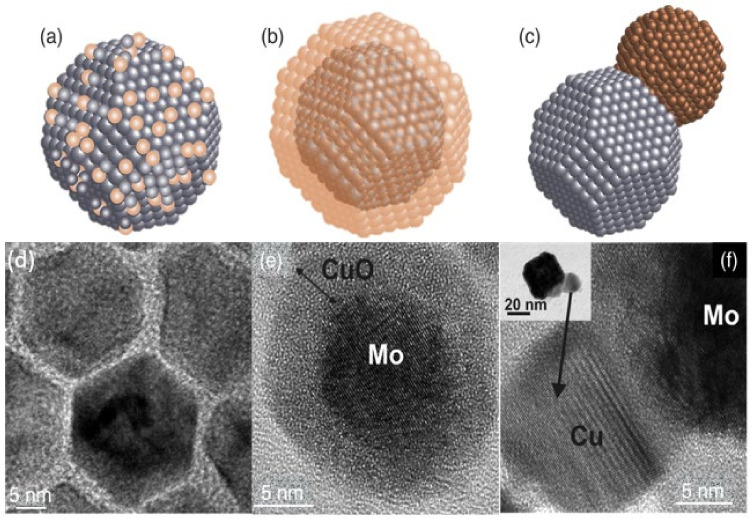
Basic structural forms of nanoparticles containing more than one element: (**a**) a uniform Pt-Ni alloy; (**b**) a Mo@CuO core–shell nanoparticle; (**c**) a Mo-Cu Janus particle. (**d**) Reproduced with permission from [22]; (**e**,**f**) reproduced with permission from [23].

Much of this discussion applies to elemental nanoparticles, but the field has moved on to nanoparticles containing more than one element. This is partly due to a fundamental interest in nanoscale interfaces but also because optimizing performance in applications requires nanoparticles containing two or more elements. Introducing just one extra element produces a rich variety of nanostructures, which can be characterized as one of three basic structural forms illustrated in Figure 1, namely, a uniform alloy (Figure 1a), a core–shell arrangement (Figure 1b) or a Janus particle (Figure 1c), in which the different elements are separated into bonded nanoparticles. There are many variations within this classification scheme; for example, a graded alloy may be considered as a core–shell particle in some cases, or the separated components in a Janus particle can contain alloys. Figure 1d,f shows TEM images of examples of these nanostructures, that is, Pt-Ni alloy particles [22], Mo@CuO core–shell nanoparticles [23] and Mo-Cu Janus particles [23]. A feature of gas-phase synthesis is that in some cases, it is possible to vary the structural motif between the basic forms illustrated in Figure 1 by changing the synthesis conditions within the source, as was demonstrated in the case of MoCu [23].

This review focuses on gas-phase synthesis of multi-element nanoparticles, with the following section describing the synthesis methods used. Section 3 provides an extensive literature review of multi-element particles produced by these methods. Section 4 discusses the structural forms, taking into account the surface energies and enthalpy of mixing of the elements. Finally, some examples of technological applications are presented in Section 5.

## 2. Gas Phase Methods for the Synthesis of Multi-Element Nanoparticles

The most common method of forming a beam of gas-phase nanoparticles is to generate a vapor of the material required in a flow of inert gas, in which it becomes supersaturated. It thus naturally condenses into nanoparticles, the size distribution of which depends on the conditions in the source, such as the supersaturation ratio of the vapor, the temperature and the pressure. The source of the vapor can be a thermal crucible [24], a laser-ablated target [25], a sputter target [26], an arc [27] or a high-voltage spark [28]. Most sources, without the inert gas flowing, are maintained at high or ultra-high vacuum, and the gas containing the clusters is passed through a skimmer and a set of differentially pumped apertures so that the final output is a beam of nanoparticles moving through high vacuum. A notable exception is the spark source, described below, which operates at atmospheric pressure and the output of which is a nanoparticle aerosol within the inert gas.

The condensation of a vapor into particles is a standard problem in classical physics and can be understood in terms of the Kelvin equation, which describes the vapor pressure of a substance, *p*, above a curved surface of radius *r* in terms of the vapor pressure of the same substance above a flat surface, *p_0_*:

(1) $$p=p_{0}e^{2\gamma v/rkT}$$

Here, *γ* is the surface tension of the condensed phase, *v* is the volume of a molecule of the material, *T* is the absolute temperature and *k* is Boltzmann’s constant. For a given value of the supersaturation ratio, *p*/*p*_0_, Equation (1) predicts a critical diameter *r_c_*, for a condensed nanoparticle to be stable, which is given by:

(2) $$r_{c}=\frac{2\gamma v}{kTlog_{e}\left(\frac{p}{p_{0}}\right)}$$

Thus, at a given temperature and supersaturation ratio, a particle with a radius greater than *r_c_* grows, while one smaller than *r_c_* shrinks and disappears. For a pure metal vapor, an enormous supersaturation ratio is required to produce condensation, since the critical radius is significantly larger than a single atom. This means that a large number of atoms have to collide simultaneously to produce a seed particle large enough to continue growing, which is termed homogenous nucleation. It is the inert gas, introduced at a higher pressure than the material vapor pressure, that brings the critical radius down to a value where homogenous nucleation can produce seed particles that are able to grow. The nanoparticle output of gas-phase sources has been observed to follow a log-normal size distribution given by:

(3) $$F\left(n\right)=\frac{1}{\sqrt{2\pi \ln\sigma }}e^{-{\left(\frac{\ln n-\ln\bar{n}}{\sqrt{2}\ln\sigma }\right)}^{2}}$$

where *n* is the number of atoms in a cluster and σ is the variance. This was also predicted by Monte Carlo modelling in the case of Cu nanoparticles forming in an Ar flow [29].

In vacuum-based sources, the critical size is usually larger than an atom, and homogenous nucleation represents a bottleneck for the initial growth. This is circumvented to some extent in sputter sources, in which the sputtered vapor is rich in dimers and larger clusters that form initial seeds. Also, in spark sources that operate at atmospheric pressure, the conditions bring the critical radius down to smaller than an atom, so condensation into nanoparticles proceeds from every atomic collision. The description so far applies to any gas-phase nanoparticle source, and in principle, all such sources can produce multi-element nanoparticles by employing alloy targets. We now focus on instruments that are designed specifically to produce heterogenous nanoparticles.

**Figure 2:**
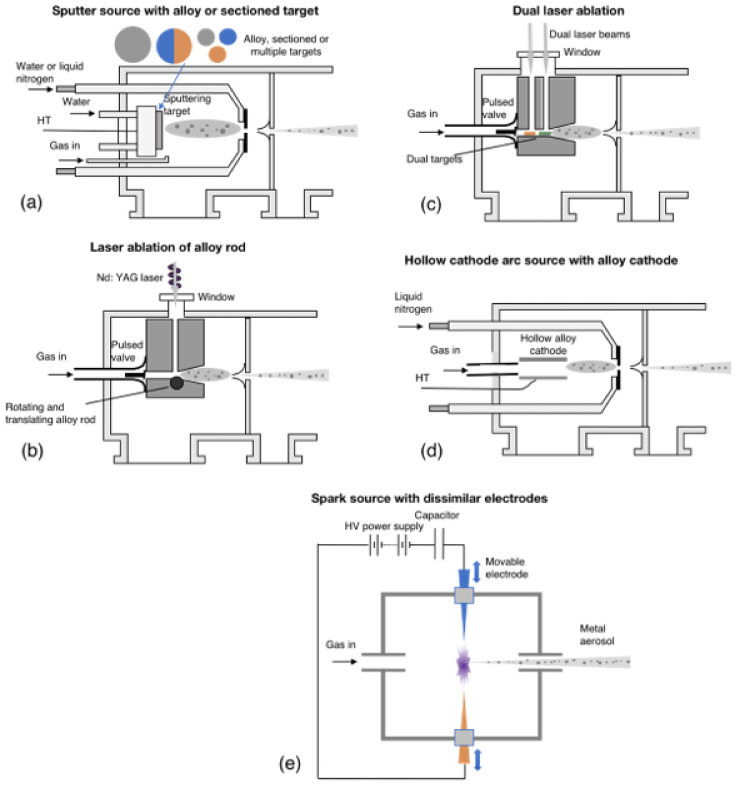
Types of gas-phase source used to produce binary metal nanoparticles using the “simultaneous” method. (**a**) A sputter source; (**b**) a dual laser ablation source; (**c**) a laser ablation source; (**d**) a hollow cathode arc source; (**e**) a spark ablation source.

The technologies developed for gas-phase synthesis of bimetallic nanoparticles date back to 1999 [30] and can broadly be characterized as “simultaneous” and “sequential”. In the former category, the materials of which the nanoparticles are to be made are simultaneously evaporated in the aggregation region and form nanoparticles with various structural motifs, which include alloy, core–shell and Janus particles [23]. Some control can be exercised over the type of binary mixture formed by varying the source conditions [23,31]. In principle, any gas-phase source can produce bimetallic nanoparticles by using an alloy as the source material, but some machines, as illustrated in Figure 2, have additional control over the overlap of the elemental plumes. For example, in the dual laser ablation source (Figure 2c), the timing of the laser pulses can evaporate one material before the other [30]. In the sputter source (Figure 2a), the final structure can be varied by using multiple independent sputter targets [32] or having a single sectioned target [23]. In order to further control the final structure, Huttel et al. developed a sputter device with three independent and movable magnetrons, which allowed even more control in the position and timing of vaporization of the different targets [33].

**Figure 3:**
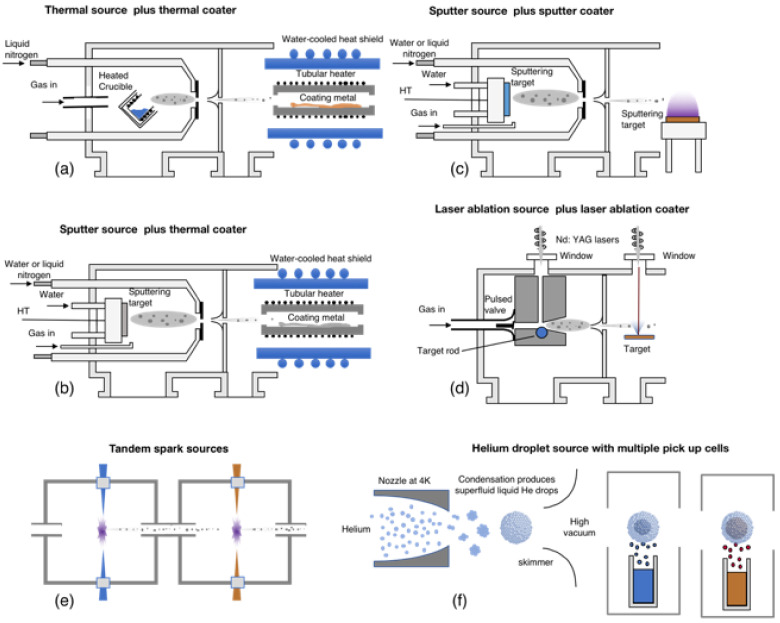
Sequential methods for producing core–shell nanoparticles. (**a**–**e**) are all “hot” coating techniques, that is, the shells are coated onto the core nanoparticles in a high energy environment in which the core nanoparticles are heated. (**f**) Core–shell particles produced by condensation of metal vapor within liquid He droplets, which is a “cold” coating technique.

In the sequential approach, which is aimed specifically at producing core–shell structures, the core nanoparticles are generated in the aggregation region of the source and then passed through a separate deposition stage to coat them with another material. Various methods, illustrated in Figure 3, have been tried, including a thermal source with a thermal coater (Figure 3a) [34], a sputter source with a thermal (Figure 3b) [35] and sputter (Figure 3c) [36] coater and a laser ablation source with a laser ablation coater (Figure 3d) [37]. Spark sources can also be placed in tandem, as illustrated in Figure 3e, for sequential coating of core nanoparticles. These can all be characterized as “hot” coating methods (see below), but Figure 3f shows an alternative gas-phase method in which the nanoparticles are grown in micron-sized liquid He droplets, which fly sequentially over metal pick-up cells [38]. The liquid He droplets are completely transparent to the thermal radiation over the pick-up cells and remain at their production temperature of 0.4 K (the He lambda point). The metal atoms that enter the droplet quickly equilibrate with the temperature bath of the droplet, and the metal–metal bonding causes the evaporation of thousands of He atoms. The large size of the He droplets, however, allows them to absorb hundreds of metal atoms before they disappear. This can thus be characterized as a “cold” method in which the core nanoparticles and any subsequent shells are formed at 0.4 K.

A naïve representation of the particles produced is shown in Figure 1b. However, the “hot” sequential methods often produce alloy or Janus particles depending on the combination of materials. One issue is that every “hot” coating method heats the core nanoparticles, and the coating is produced in a high energy environment. For example, with the thermal coaters, core nanoparticles are heated within a few nanoseconds to the temperature within the coater (typically 1000 °C). The time of passage through the coater is typically ~1 ms, so the core nanoparticles spend the entire time within the coater at the high temperature. This automatically restricts the method to coating with materials with a higher vapor pressure than the core nanoparticles; otherwise, these will evaporate within the coater. Sputter coating the shell also provides a high-energy environment generated by the plasma required to vaporize the target, which is also true for the spark source. On the other hand, using laser ablation to provide a coating exposes the core nanoparticles to the laser, which heats them. Thus, in all these methods, the coating occurs in an environment in which there is plenty of energy available for diffusion to allow the particles to adopt the most stable structure thermodynamically, which may not be the core–shell arrangement. Even the “cold” coating method shown in Figure 3f doesn’t necessarily produce simple core–shell particles, as it has been shown that multiple cores can form within the liquid He droplet [39]. Because of the very rapid cooling of atoms entering the liquid He droplets, however, this technique can produce metastable structures not observed with the other synthesis methods (for example, disordered Cr clusters with a significant ferromagnetic moment of 1.83 µB/atom) [40].

It is clear that whichever method is used, nanoparticles with a core–shell morphology can be formed only with certain combinations of materials, and even with a suitable combination, only for certain particle sizes (as discussed in Section 4). It is demonstrated in later sections, however, that the sequential approach has greater utility in producing the required core–shell structure.

In any case, the final morphology of the nanoparticle can be understood and even predicted by computational methods. Although a thorough description of these methods is beyond the scope of this review, the Monte Carlo method [41,42] along with molecular dynamics [43,44,45] calculations should be referenced, as they play a key role in comprehension of the formation and morphology of heterogeneous nanoparticles.

## 3. Results

There has been a large number of studies of multi-element nanoparticles produced in the gas phase, and it was not practical to describe a case study of each one. Instead, this section presents a summary overview of all systems, and a discussion of general conclusions that can be drawn is deferred to Section 4. This section is subdivided into two-element nanoparticles (the vast majority) and three-element nanoparticles.

### 3.1. Two-Element Nanoparticles

**Figure 4:**
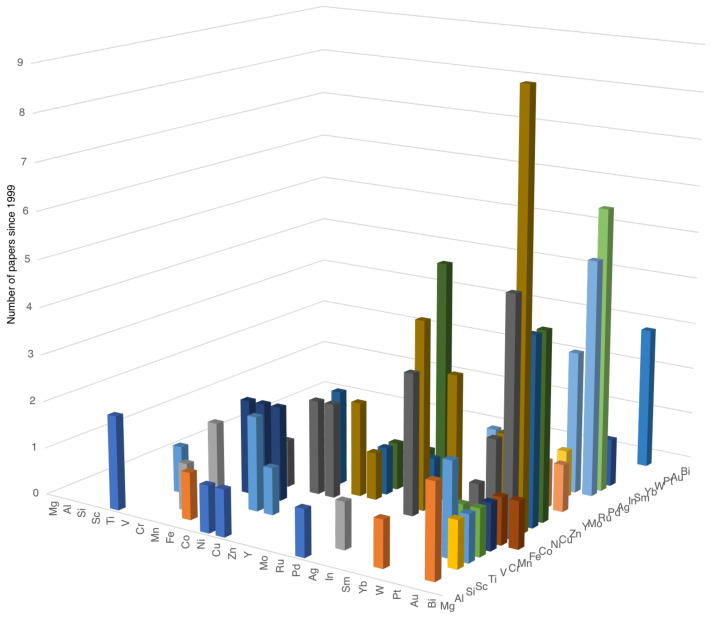
Two-element nanoparticle systems reported since 1999. When looking for a particular combination, search along the line of the lighter element.

The first reports of two-element nanoparticles produced in the gas phase emerged in 1999 from the group of Peter Lievens in Leuven, who used a dual laser ablation source (Figure 2c) to generate small AuCu, AuAl, AuY and AuIn clusters [30]. These contained less than 100 atoms, and the interest was in determining the effect on the pattern of magic numbers, or especially stable clusters, of adding dopant atoms of different elements to Au. Since then, the field has grown to study nanoparticles with combinations of elements ranging from Mg to Bi; the large variety and number of systems reported is illustrated in Figure 4. This chart was compiled by putting the lightest element first in every pair, so when looking for a particular combination, one should search along the line of the lighter element.

The element that has been combined with the largest number of additional elements is Au, and the most studied system is CoAu. In general, the systems with the largest numbers of reports are those of most interest in applications.

Table A1 in Appendix A presents all the two-element nanoparticles studied, ordered by year, showing the gas-phase method used to produce them, their size and their deduced structural arrangement (alloy, core–shell or Janus). Table 1 below presents a summary of the two-element systems reported to date presented in a more condensed form, which we believe has more utility for researchers. The left column has elements listed by increasing atomic number; for each is listed every element it has been put in combination with it, again, in increasing atomic number along with the relevant references. Also listed in the table for each combination are the structural forms of the nanoparticles reported; the bulk surface energy difference, obtained as described in Section 4 and the bulk enthalpy of mixing, which is given for the 50/50 alloy or as close as is available. These data are presented in preparation for the discussion in Section 4. The +/− sign indicates whether the elements are miscible (−) or immiscible (+); in cases where the enthalpy was not available, this is denoted NA. In cases where there was an observed core–shell structure, the surface energy difference is given by the shell surface energy minus the core surface energy, whereas for other structures it is listed as a magnitude. In all the tables, oxygen in oxidized nanoparticles is not considered as a separate element, and only the metallic or semiconducting elements are listed. In addition, the term “sequential” is reserved for coating materials that are deposited in the high vacuum region of a vacuum source, or in the case of spark sources by separate downline spark electrodes as illustrated in Figure 3e.

A significant proportion of the earlier studies focused on very small clusters of fewer than 100 atoms and determined the effect on the magic number sequence of the pure material of adding dopant impurities of other elements [30,31,61]. Indeed, the observation of magic numbers in gas-phase clusters preoccupied much of the community, as mass spectra of photoionized clusters showed peaks indicating especially stable clusters with a given number of atoms. For many metals, such as Au, the stability peaks represented filled electronic shells of delocalized electrons contained in the spherical potential of the whole particle [140]. Thus, in the case of Au, peaks should be observed for clusters containing 2, 8, 18, 20, 34, etc. electrons, or, for singly ionized clusters, abundance peaks in the mass spectrum should occur at Au_3_^+^, Au_9_^+^, Au_19_^+^, etc. It was found that doping with other elements generally preserved the shell closures and magic numbers if the difference in valence electrons contributed by the dopant was taken into account.

For larger particles containing mixtures of elements, the equilibrium structures can in most cases be understood in terms of the energy balance between the enthalpy of mixing and the surface energy, both of which are size dependent. In a simple analysis, if the enthalpy of mixing is negative, an alloy is favored; however, if there is a large surface energy difference between the two elements, then below a critical size, a core–shell or Janus configuration has lower energy.

A clear-cut example of applying the rules is CoAu, for which there is a positive enthalpy of mixing and a significant surface energy difference (lower for Au). Thus, in all studies reported [86,102,103,104,105,106,107], the nanoparticles were in the Co@Au core–shell configuration, except in one case [105], where they showed a Janus structure. On the other hand, for CoSm, there is a large negative enthalpy of mixing and a smaller surface energy difference; thus, in every case, the nanoparticles were alloys [37,97,98]. However, as the size decreases, the surface energy per atom increases and the enthalpy of mixing decreases, so a positive enthalpy for immiscible elements can become negative [141], favoring an alloy in sufficiently small particles. This was observed in nanoparticles of MoCu, which formed an alloy from metals that are immiscible in the bulk [23].

The energy arguments apply to equilibrium structures; however, the synthesis methods under examination can synthesize particles in non-equilibrium arrangements. Whether or not these are maintained depends on the energy barrier to overcome in order to obtain the equilibrium configuration. The clearest example of this is AgAu nanoparticles prepared by the He droplet method (Figure 3f) [136], in which the cluster formation and shell deposition all occur below 4 K. With this combination of elements, the alloy is favored, but the method was able to prepare nanoparticles of Ag@Au and even Au@Ag (in which the higher-surface-energy material was in the shell) and maintain these structures after deposition onto a substrate at room temperature. In both cases, the nanoparticles’ structures transformed to the alloy after annealing to 573 K. A more detailed description of the expected structures and comparisons with experiment are given in Section 4.

### 3.2. Three-Element Nanoparticles

Since 2005, reports of three-element nanoparticles produced by the gas-phase techniques described above have started to appear [141]; the systems studied so far are listed in Table 2. The desire to include more elements within nanoparticles is driven to a large extent by technological requirements. For example, in the case of medical applications, it is possible to produce a nanoparticle that can execute several diagnostic and therapeutic functions simultaneously, as described in Section 5. When moving to three elements, there are additional possibilities for the internal structure, including ternary alloys, an alloy core and elemental shell, a Janus core and an elemental shell, an elemental core and two elemental shells and an elemental core with an alloy shell. Table 2 shows that all but the latter configuration have been observed. There are also more complex arrangements (for example, multiple cores and graded alloys without sharp interfaces), as described below.

**Figure 5:**
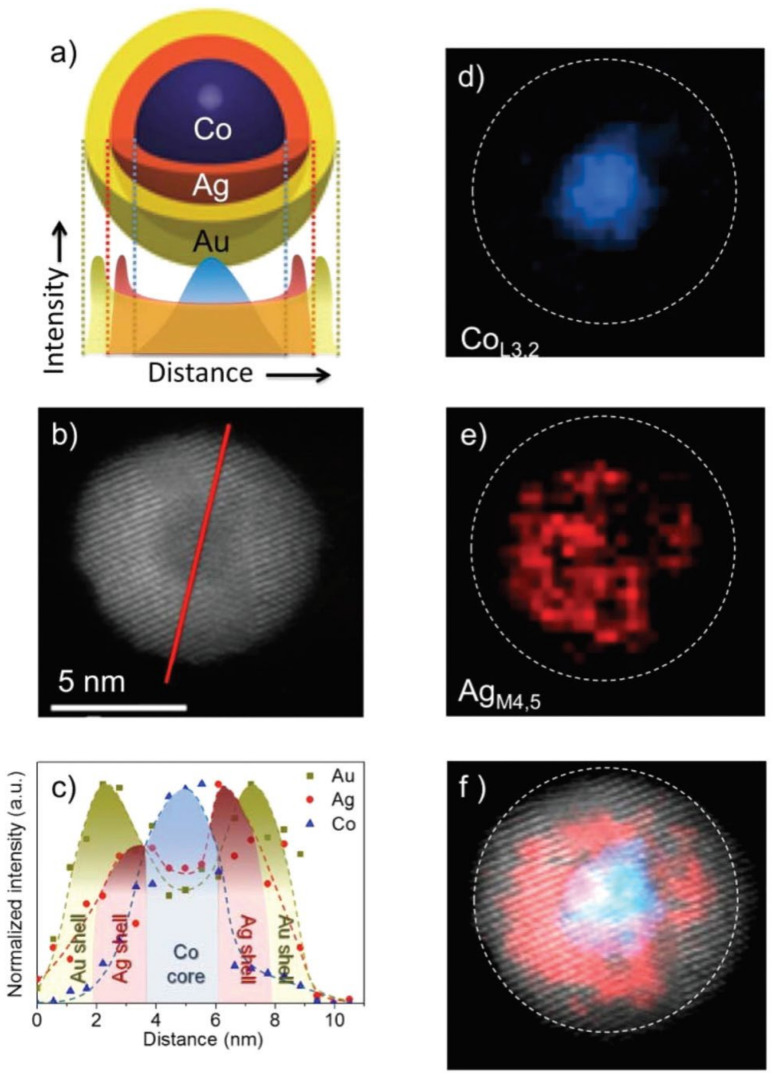
(**a**) Representation of the Co@Ag@Au structure together with the expected EDX intensity profiles. (**b**) TEM representative image of a Co@Ag@Au nanoparticle. (**c**) EDX line scan performed at the Co, Ag and Au peaks along the line depicted in (**b**). (**d**) EELS compositional analysis for the Co L_3,2_ edge. The dashed line represents the outer limit of the nanoparticle. (**e**) EELS map for the Ag M_4,5_ edge. (**f**) STEM image together with the corresponding Co and Ag EELS concentration maps superimposed. Reproduced with permission from [103].

The work on (FeCo)@Ag and (FeCo)@Au [142] pursued a non-oxidized magnetic core with the highest magnetization transition metal alloy (FeCo) within a biocompatible shell that prevented oxidation, which was achieved. The Huttel group in Madrid developed a sputter cluster source with multiple movable targets (see Figure 2a) and have used it to produce ternary alloy nanoparticles [33] and the clearest demonstration yet of a core–shell–shell system, that is, the Co@Ag@Au particles shown in Figure 5 [103]. The schematic of the organization of elements within the nanoparticles is shown in Figure 5a, along with the signals that would be expected from an energy-dispersive X-ray (EDX) line scan across the particle. A TEM image of a single three-element nanoparticle is shown in Figure 5b, and the red line indicates the scan line for the EDX data shown in Figure 5c. This shows the expected variation for the signal and clearly indicates the separation of the three elements for the proposed structure. Further confirmation of the elemental organization of the nanoparticle comes from the electron energy loss spectra (EELS). When tuned to the Co L_2,3_ edge (Figure 5d), just the core is visible whereas focusing on the Ag M_4,5_ edge (Figure 5e) shows the Ag distributed as a shell around the Co core. The core–shell structure is yet more evident in Figure 5f, where the Co and Ag EELS images are superimposed.

A similar three-target sputter source was used by the “Nanoparticles by Design” group in Okinawa to produce SiFeAg nanoparticles. They found that the nanoparticles had one or multiple cores of FeAg Janus particles with a shell of Si. This structure can be explained by the large surface energy difference between Fe and Ag; in every report of two-element FeAg nanoparticles, a Janus particle has been observed (see Table 1). In addition, two-element SiAg particles have been observed to have either a core–shell or Janus structure with multiple cores (see Table 1).

**Figure 6:**
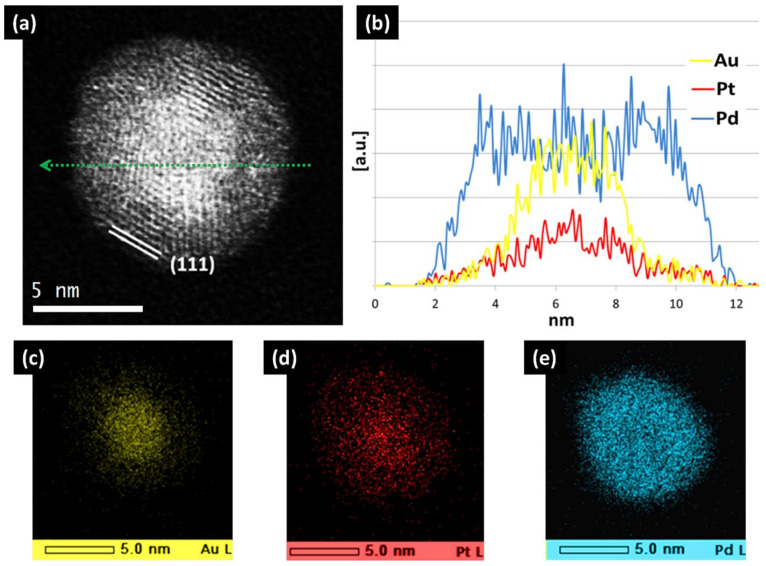
(**a**) HR-STEM-HAADF micrograph of a multifaceted PdPtAu nanoparticle displaying fivefold symmetry and (**b**) its corresponding EDS line scan across the nanoparticle diameter (dashed green line), indicating the Au/Pt-rich core@Pd-rich shell configuration. (**c**–**e**) Local elemental mapping by EDS verifying the core−shell structure (the intensity corresponds to counts). Reproduced from [41].

On the other hand, the PdPtAu particles show a graded alloy with a AuPt-rich core and a Pd-rich shell, as illustrated in Figure 6. This is unexpected from simple energy balance arguments based on bulk values, as the enthalpy of mixing for PtAu is positive, whereas it is negative for both PdPt and PdAu. In addition, the surface energy of Pd is higher than the average value for PtAu, so it would not be expected to accumulate in the shell. This is a clear demonstration that theses energy values are significantly different in nanoscale systems, as discussed below.

## 4. Discussion

One of the most important considerations when synthesizing multi-element nanoparticles is which of the three basic structures illustrated in Figure 1 they will form. The equilibrium structure is determined by minimizing the particle energy, considering the surface energies of the materials and the enthalpy of mixing and bearing in mind that both of these are size-dependent. It is also possible with some methods to form non-equilibrium structures with a sufficient energy barrier separating them from the equilibrium arrangement to keep them stable at moderate temperatures, as discussed below.

### 4.1. Surface Energies—Structures for Nanoparticles of Immiscible Elements

**Figure 7:**
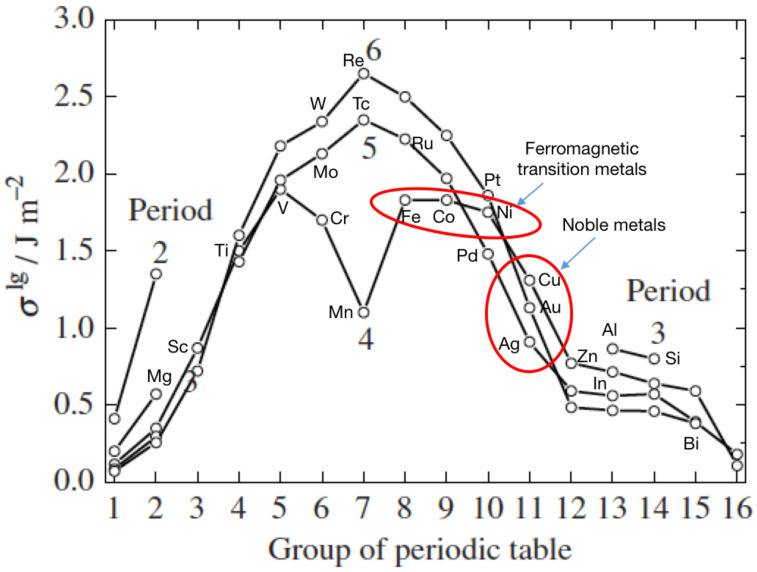
Value of surface tension (surface energy) of liquid metals at their melting points, with the elements appearing in Table 1 marked. Reproduced with permission from [145].

We begin by considering the surface energy and assume that the elements are immiscible (at the size of interest) so that the structure is entirely determined by the surface energy difference of the materials. In an isotropic system, the surface energy is the same as the surface tension, *σ*, given by:

(4) $$\sigma ={\left(\frac{\partial U}{\partial A_{s}}\right)}_{S,V,n},\;({\mathrm{Jm}}^{-2})$$

where *U* is the internal energy, *S* is the entropy and *n* is the number of moles in the surface. A liquid surface is an isotropic system; it is useful to consider this first, as it is possible to use this knowledge to determine some trends. Small clusters of simple metals can be considered to be liquid particles, as demonstrated by their stability being determined by electronic shell filling within a spherical potential as opposed to geometrical packing [140]. The surface energy values for liquid metals at their melting points is plotted in Figure 7, and it is observed that, apart from the anomaly at Mn, the same trend with group number is followed for every period in the periodic table. The elements relevant to this review are marked in the figure.

For a solid, the surface energy, γ, is defined by:

(5) $$\gamma ={\left(\frac{\partial G}{\partial A_{s}}\right)}_{T,p}\;({\mathrm{Jm}}^{-2})$$

where *G* is the Gibbs energy, *A*_s_ is the area of the surface, *T* is the temperature and *p* is the (environmental) pressure, which is zero for a particle in a vacuum. The surface energy is given by the number of bonds per unit area and depends on the strengths of the bonds. In a solid single crystal, the surface energy is anisotropic and depends on the alignment of the surface exposed, and thus for particles, which present a number of different crystal planes at the surface, a weighted average must be taken. For a (111) plane, there are six nearest neighbors in the plane, three above and three below. Hence, three bonds are broken for each surface atom when the crystal is cut along (111). For the (100) and (110) planes, there are four and six broken bonds, respectively, and thus, taking only nearest neighbors into account, the surface energies for these planes are larger. Thus, to determine the surface energy of a nanoparticle, we need to know the number of atoms in each crystallographic plane at the surface.

For metal nanoparticles, the crystal structure produces polyhedra, the shape of which is often predicted by the Wulff construction [146]. This was formulated for macroscopic crystals and strictly applies only to large particles in which edge and vertex effects are negligible. The method was developed to describe the shape of gemstones, for which the equilibrium shape is obtained after eons at extreme temperatures and pressures. In nanoparticles, however, the equilibrium shape can emerge in fractions of a second, and so it predicts the correct shape for a number of fcc and bcc metals. There are cases, however, when during synthesis, there is insufficient energy and/or time available for the equilibrium shapes to emerge, as is discussed below. The majority of materials and nanoparticle sizes of interest are within this class, so the focus for the rest of the report is on polyhedral shapes predicted by the Wulff construction.

**Figure 8:**
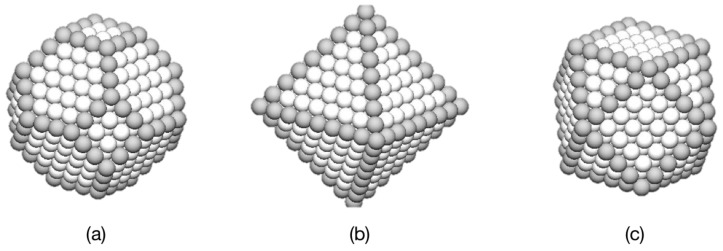
Equilibrium and “close to equilibrium” morphologies for nanoparticles of fcc metals. (**a**) A 586-atom polyhedron conforming to the Wulff construction. (**b**) A 489-atom octahedron containing only (111) faces. (**c**) A 561-atom cuboctahedron produced by truncating an octahedron by a cube. Reproduced with permission from [5].

In this scheme, the equilibrium polyhedron is constructed so that the distance from the center of the nanoparticle to a face is proportional to the specific surface energy of that face. For an fcc crystal, this procedure produces a polyhedron consisting of eight (111) faces and six (100) faces, as shown in Figure 8a. Sometimes, departures from the ideal shape can be produced by specific conditions and bonding anisotropies that lead to “close to equilibrium” shapes. For example, another possibility for fcc metals is an octahedron containing of only low specific energy (111) surfaces, shown in Figure 8b. This does, however, have a larger total area for a given number of atoms, and in some cases the energy can be reduced by truncating it by a cube to produce a cuboctahedron (Figure 8c) despite the introduction of higher-specific-surface-energy faces. The polyhedra shown in Figure 8 are completed when they contain a specific number of atoms, *N*, known as magic numbers. For each shape, there is a set of magic numbers corresponding to the completion of a polyhedron with a given number of atomic shells, *K*. For example, for the octahedron:

(6) $$N=\frac{1}{3}\left(2K^{3}-K\right)$$

giving magic numbers of 6, 19, 44, 85, 146, 231, 344, 489, etc. Once a shell is complete, it is expected that growth of the next layer should begin on the (111) faces.

**Figure 9:**
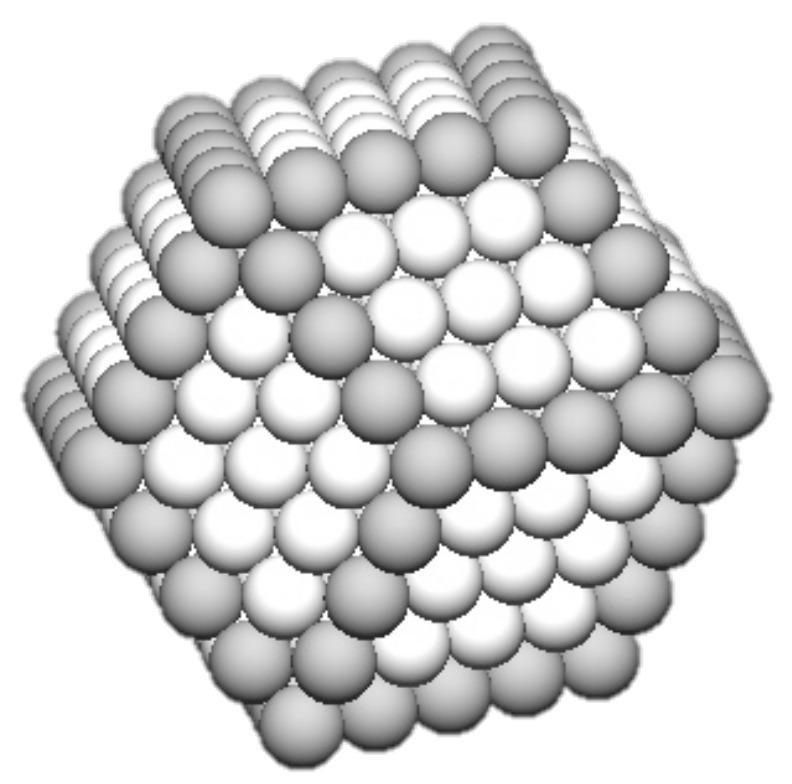
Equilibrium morphology for nanoparticles of bcc metals. A 369-atom dodecahedron with 12 identical faces. Reproduced with permission from [5].

In the case of particles with a bcc lattice (for example, Fe), applying the Wulff construction produces a dodecahedron made up of 12 identical faces, as shown in Figure 9. In this case, shell filling numbers are given by:

(7) $$N=4K^{3}-6K^{2}+4K-1$$

giving *N* = 15, 65, 175, 369, 671, etc. Experimentally, it has been found that bcc metals such as Fe or Mo often form cubes; this is because the surface energy of six (110) faces is very close to the weighted average of the faces on the Wulff construction of the same volume.

**Figure 10:**
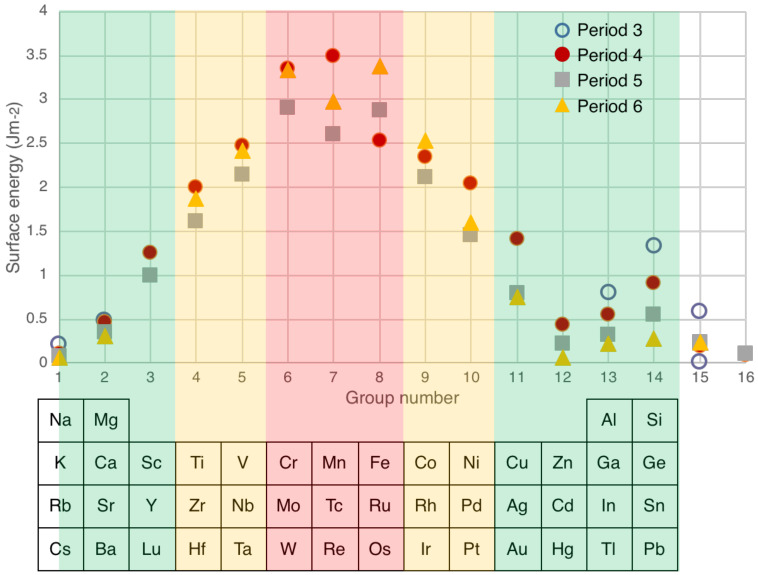
DFT calculations of surface energies of solid particles with a Wulff morphology [147]. The surface energies are banded into low, medium and high values (green, amber and red, respectively, following [89]), with the relevant portion of the periodic table shown below.

Knowing the crystalline planes presented at the surface of a nanoparticle, it is possible to perform density functional theory (DFT) calculations to obtain the surface energies of each face. There is now a full database available for most of the solids in the periodic table [147] as well as a user-friendly interface called “*Crystalium*” [148] that returns the weighted average of the surface energy, assuming a Wulff construction, for the particle. The surface energies of period 3, 4, 5 and 6 solids of the periodic table, as calculated by *Crystalium*, are plotted in Figure 10. In comparison to Figure 7, which is for liquid metal surfaces, it is clear that the same trend is followed but the surface energies are around 40% higher, as would be expected for a solid.

Given the similarity of surface energy values for specific groups across all periods, it makes sense to follow a scheme suggested by Koten et al. [89] and band the surface energies vertically in the periodic table into low, medium and high values. This is illustrated in Figure 10, where low (green), medium (amber) and high (red) surface energies are projected onto the relevant parts of the periodic table. Thus, for immiscible combinations of elements, red materials form cores in a core–shell structure with amber and green materials, while amber elements are at the core of a core–shell structure with green elements. Picking out only the immiscible combinations of elements listed in Table 1, the core–shell particles Ti@Mg, Fe@Cu, Fe@Au, Co@Ag, Co@Au, Ni@Cu, Ni@Ag, Ni@Au and Mo@Cu all conform to this simple classification. The combinations W@Fe and Cu@Ag both include elements of the same color code, but Figure 10 shows that the surface energy differences within the bands still give the lower surface energy material in the shell. Some of the combinations given above have also been observed to form alloy nanoparticles, but these are not anomalies, since, as discussed below, it is possible for the enthalpy of mixing to become negative below a critical size. Also, Janus structures have been seen for some of the combinations, as certain shapes of Janus particles can have a lower energy than the core–shell structure.

The only true anomalies are Ag@Si, Fe@Cr and Co@Cr. In the case of Ag@Si the nanoparticles do not have a simple core–shell structure but contain multiple cores. This can be seen as a phase separation of the immiscible elements within the particle. In the two reports of Fe@Cr [34,71], in one, the nanoparticles were prepared in a spark source with hydrogen included in the gas flow [71], which can significantly affect the surface energies. The other was for very small (2.8 nm) nanoparticles produced in a UHV source, and as discussed below, the surface energies can depart significantly from the values shown in Figure 10 for such small particles. For the Co@Cr case, the EELS chemical maps of the nanoparticles indicated that the shell was Cr oxide, and the calculated surface energies of various low index surfaces of the native oxide Cr_2_O_3_ are lower than those of the metal [149].

The entire discussion so far has been conducted using bulk values of surface energies, but it is possible that the surface energy of nanoparticles can be changed due to the mechanical strain in curved surfaces. For example, the surface energy of Ag nanoparticles with diameters in the range of 17–19 nm was measured to be 7.2 Jm^−2^ [150], compared to 0.79 Jm^−2^ for the bulk. In the case of Pd nanoparticles with diameters in the range of 1.4–5 nm, the measured surface energy was 6 ± 0.9 Jm^−2^ (cf. the bulk value of 1.48 Jm^−2^) [151]. However, values reported through both theory and experiment vary widely, and how surface energy depends on particle size is still under debate. It was argued recently that the strain induced by curvature is compensated by the decrease in nearest neighbors, and the variation with size is therefore rather weak [152]. Indeed, it was stated that, unless reliable experimental measurements are available for the size of interest, the bulk value should be used. The other relevant point is that in a core–shell nanoparticle, since the core and shell are of a similar size, the curvature might be expected to have a similar effect on the surface energies. This would be less valid for very small nanoparticles, which could lead to anomalies such as that discussed above for 2.8 nm-diameter Fe@Cr nanoparticles. For immiscible materials, using the bulk values of surface energy calculated by *Crystalium* successfully predicts the majority of core–shell structures; therefore, these values continue to be used in this review. Table 3 is a list of surface energies for the elements listed in Table 1, obtained using *Crystalium*, and the value for the liquid metal, obtained from Figure 7, is shown for comparison.

### 4.2. Enthalpy of Mixing—Structures for Nanoparticles of Miscible Elements

**Figure 11:**
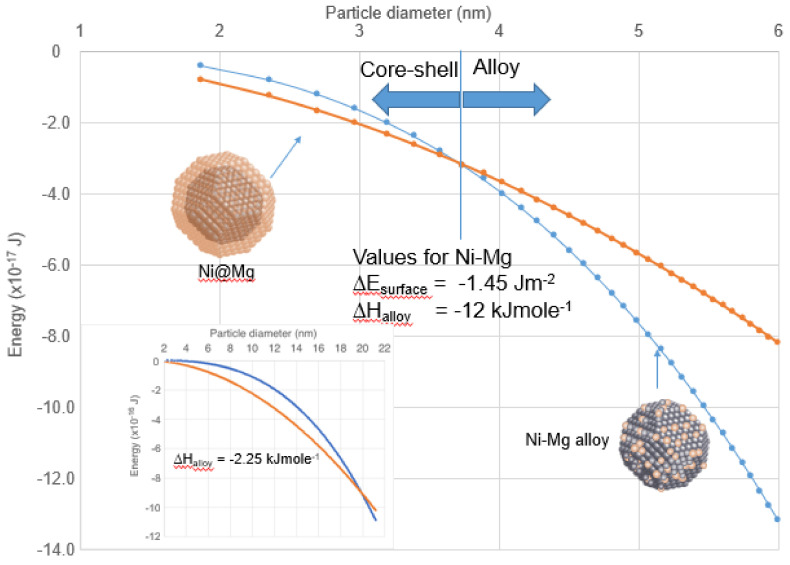
The size dependence of the enthalpy of mixing, assuming the molar value stays constant (blue line), and the difference in surface energy between the core–shell and alloy structures (red line), with values appropriate for bulk NiMg with a 50/50 concentration. It is assumed that the surface energy of the alloy is the average of the Ni and Mg values. Below a critical size (3.7 nm) the core–shell structure has lower energy. The inset shows the calculation repeated for a reduced enthalpy to shift the critical size to 20 nm core–shell particles observed experimentally [48].

In element combinations for which the enthalpy of mixing is negative, the formation of an alloy is favored in the bulk. In a nanoparticle, as the size decreases, the enthalpy scales with the particle diameter cubed, assuming the molar value remains constant, which is not the case (see below), but is assumed for now. The total surface energy, however, scales with the diameter squared so that, even in systems with a negative enthalpy of mixing, if there is a significant surface energy difference between the elements, the core–shell configuration has the lower energy below a critical particle diameter. This is illustrated in Figure 11, which shows a calculation of the enthalpy of mixing (negative) and the surface energy difference (negative) between the core–shell configuration and the alloy with values appropriate to bulk NiMg with a 50% atomic concentration of Ni. In this calculation, the surface energy of the alloy was assumed to be the average of the two elements, and the molar enthalpy and surface energies were constant with size. Below 3.7 nm, the core–shell structure is favored.

The system Ni-Mg was observed to form core–shell nanoparticles, but the structure was identified in particles with a diameter of around 20 nm, which implies a significantly reduced enthalpy. The inset in Figure 11 shows that the magnitude of the enthalpy of mixing needs to be reduced to −2.25 to shift the critical diameter to 20 nm. There have been several other reports of core–shell nanoparticles in combinations with negative enthalpies of mixing, including Cu@Mg, Pd@Mg, Fe@Al, Al@Yb, V@Pt, Fe@Zn and Ag@Au. In every case, the particles were larger than the critical diameter obtained using the simple calculation above. This indicates a significantly altered enthalpy from the bulk values shown in Table 1.

**Figure 12:**
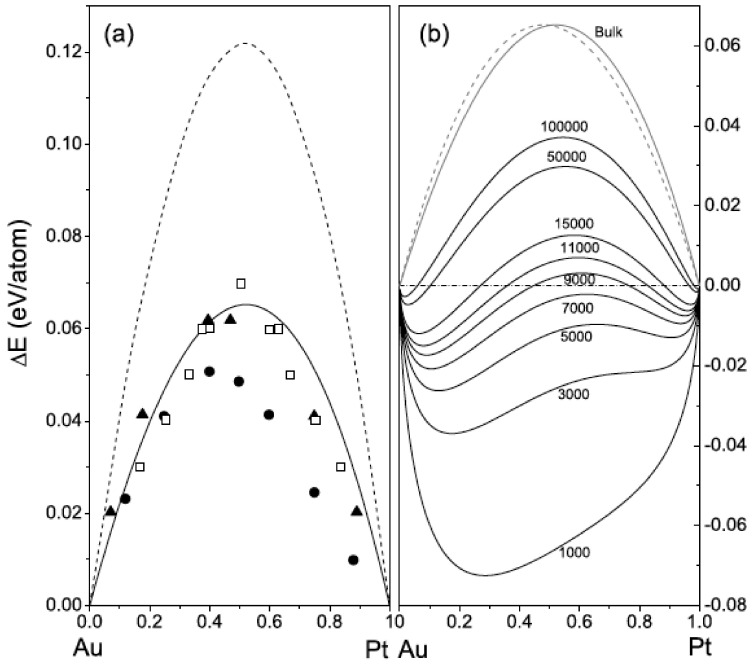
(**a**) The enthalpy of mixing for the bulk Au-Pt system, calculated using an embedded atom method as a function of Pt concentration, showing the expected peak at Au_50_Pt_50_ (solid line). The other data are previous calculations using an EAM model (dashed line), an LMTO model (full circles) and the Miedema theory (open squares) as well as experimental data (full triangles). (**b**) Size and composition dependence of the enthalpy of mixing, showing a transition to negative values in the 50/50 mixture for particle sizes below 7500 atoms (6.2 nm). At low Pt concentrations, the critical size is larger. Reproduced with permission from [141].

A study of the size effect on enthalpy of mixing of Au-Pt was conducted by Xiao et al. [141] using an analytic embedded atom model; it showed a strong size dependence. In the bulk, the metals are immiscible, and Figure 12a shows the expected behavior in a macroscopic system with a peak at around the Au_50_Pt_50_ composition. Having tested the model, the calculation was repeated for particles of different sizes; the results are shown in Figure 12b. For particle sizes below around 7500 atoms (6.2 nm), the enthalpy becomes negative for the 50/50 mixture. For dilute Pt concentrations, the critical size is larger. Thus, in element combinations with a positive bulk enthalpy of mixing, which favors a core–shell structure, as the particle size decreases, it is possible for the alloy to become the lower energy state below a critical size. This is the opposite behavior to that described above.

**Figure 13:**
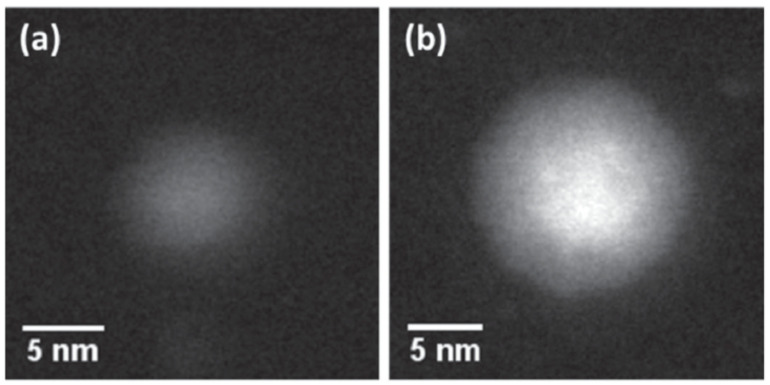
Demonstration of the transition from a core–shell to alloy structure with decreasing size for W@Fe nanoparticles. (**a**,**b**) HAADF-STEM images of a 6 nm and a 15 nm Fe-W nanoparticle. The smaller particle shows no core–shell contrast. Reproduced with permission from [89].

This transition has been observed in the case of the Fe-W system. In the bulk or in large particles, there is a small, positive enthalpy of mixing and a significant surface energy difference favoring the core–shell structure, but small particles have been seen to form the alloy, as demonstrated in Figure 13 [89]. In [89] the critical size for the transition from the core–shell to the alloy structure was determined to be around 8 nm.

### 4.3. Core–Shell vs. Janus Structures

**Figure 14:**
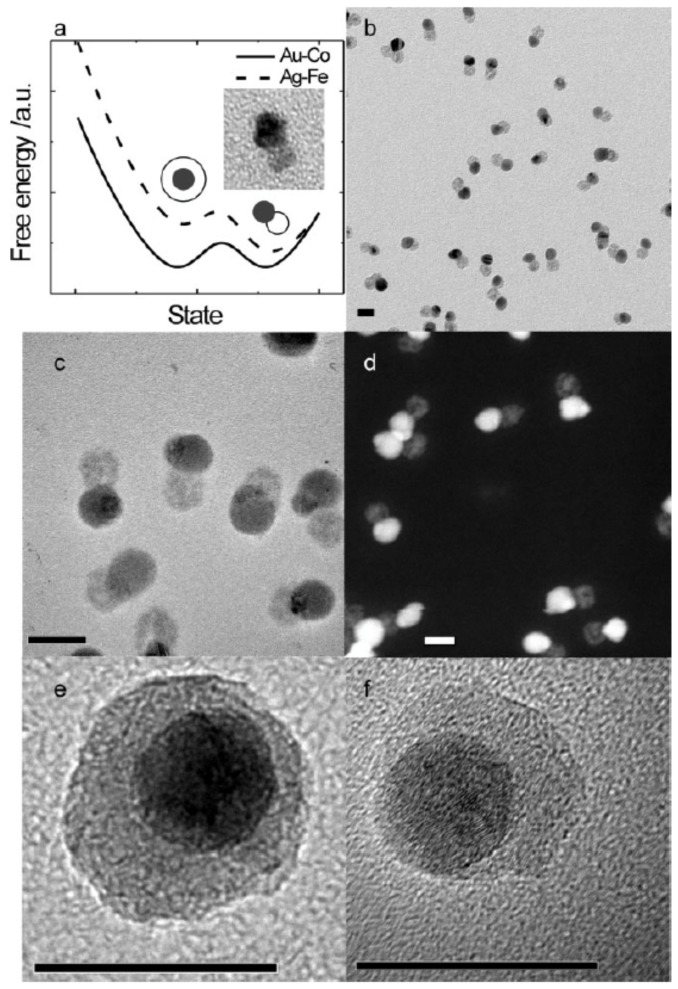
(**a**) Schematic diagram of the local energy minima separating the core–shell and Janus structures for Co-Au and Fe-Ag, with the inset showing one of the rare Co-Au Janus particles. (**b**) Wide field TEM image showing the common occurrence of Fe-Ag Janus particles. (**c**,**d**) Higher-magnification BF TEM and HAADF images of Fe-Ag nanoparticles, respectively. (**e**,**f**) Particles transitioning from the core–shell to the Janus structure. All scale bars are 20 nm. Reproduced with permission from [86].

Janus structures are only expected in particles containing immiscible metals. The only report of a Janus structure in a combination with a negative enthalpy of mixing (Mn-Fe [71]) dealt with oxidized particles. Considering immiscible materials only, both the core–shell and the Janus structures have lower energy than the uniform mixture, and for many elemental combinations, the difference is quite small. Thus, there are two local energy minima separated by a barrier, and during the formation of the nanoparticle, the height of the barrier and the valley energy in the two minima determine which structure is more likely. A study by Xu et al. [86] of Co-Au and Fe-Ag nanoparticles produced by a sputter source employing composite targets (Figure 2a) showed that, under normal operating conditions, Co-Au was much more likely to form core–shell nanoparticles. On the other hand, Janus particles were observed frequently when employing an Fe-Ag target. The authors ascribed this difference to the schematic energy diagram shown in Figure 14a, which shows a lower energy barrier to form the Janus structure in the Fe-Ag system. The inset in Figure 14a is a TEM image of one of the rare Co-Au Janus particles. On the other hand, Figure 14b shows how commonly Fe-Ag Janus particles occur in a wide field view, with higher magnification images displayed in Figure 14c,d. It takes time for the structure to form within the “hot” part of the source, so the authors caught a couple of examples of Fe-Ag nanoparticles transitioning from the core–shell to the Janus structure that were ejected before the process was complete. These are displayed in Figure 14e,f.

**Figure 15:**
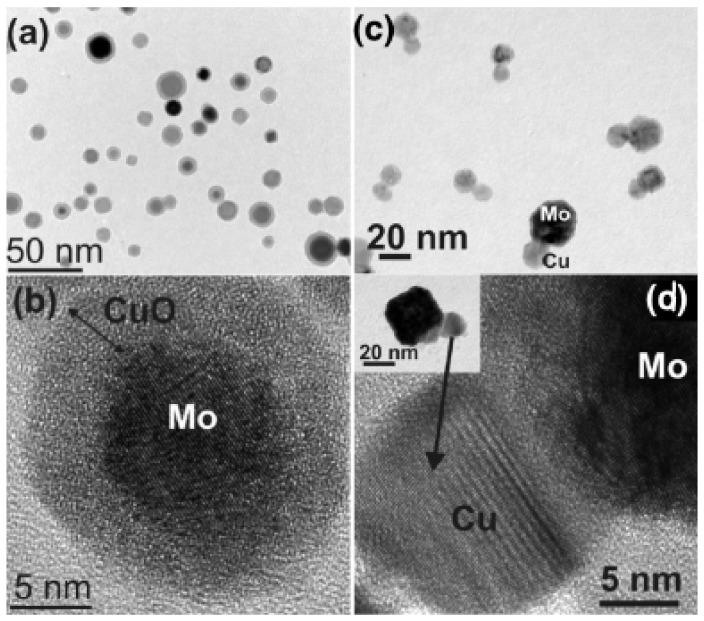
(**a**,**b**) TEM images of Mo@Cu core–shell nanoparticles produced by a sputter source with a sectioned target (Figure 2a) and an Ar flow rate of 100 sccm. The oxide forms when the particles are removed from the source to obtain the TEM images. (**c**,**d**) TEM images of Mo-Cu Janus particles produced by employing an Ar flow rate of 150 sccm. Reproduced with permission from [23].

This implies that changing the source conditions allows one to choose between the core–shell and Janus structures, as also demonstrated by Krishnan et al. [23] for Cu-Mo nanoparticles (Figure 15). These were produced using a sputter source with a sectioned target (Figure 2a). Figure 15a,b is low and high magnification TEM images of core–shell Mo@Cu nanoparticles, respectively, while Figure 15c,d shows similar images of Mo-Cu Janus particles. The only difference between the two sets of samples lay the gas flow rate in the source, which determined the particle residence time in the “hot” region of the source. The same work also found suitable conditions for the formation of CuMo alloy nanoparticles, presumably due to the enthalpy of mixing becoming negative at the scale of the particle size, as discussed in Section 4.2 above.

Langlois et al. [44] carried out a study of elemental Cu and Ag nanoparticles deposited separately onto a substrate at a sufficiently high temperature to promote diffusion between these two immiscible elements. They found that the coalescing particles formed asymmetric core–shell structures similar to those shown in Figure 14e,f, with the asymmetry depending on the Ag/Cu ratio. For a sufficiently high proportion of Ag, the full Janus structure was formed. The findings were confirmed by DFT calculations.

### 4.4. Non-Equilibrium Structures

Section 3 already alluded to non-equilibrium structures that can be stabilized at room temperature, of which there are several examples. In most gas-phase sources, nanoparticles are produced in a “hot” zone containing a mixture of the elements involved and the bath gas. The “hot” is in quotes because the target area is heated only in the case of thermal or laser sources and not in sputter, arc or spark sources. However, in the latter cases, there is a large amount of energy available from the sputtering ions or electrons in the discharge. Nucleation starts immediately above the target and is homogenous with all the elements included. Particle growth continues until the density of metal vapor becomes too small for a significant number of collisions. During growth, the seeded nanoparticles move out of the hot zone while being cooled by the bath gas; whether the equilibrium structure has time to form depends on the energy landscape around the minimum and the time the particle spends in the hot zone. This is why structures can be controlled by changing the gas-flow rates and the power used to produce the vapor.

**Figure 16:**
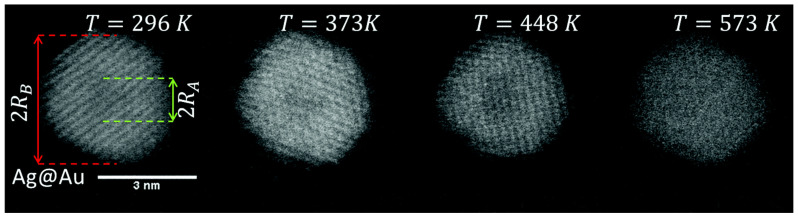
HAADF images of a single Ag@Au nanoparticle produced by the He droplet method (Figure 2f) during heating. The as-deposited nanoparticle showed a core–shell structure because the HAADF intensity was proportional to z^2^. At 573 K, the core–shell structure disappeared, and the nanoparticle formed the alloy, which is the expected equilibrium structure. Reproduced with permission from [136].

An exception to these conditions is the He droplet source (Figure 2f), in which all the particle growth occurs inside liquid He droplets at a temperature below 4 K. In principle, this method, should readily be able to produce out-of-equilibrium structures, as demonstrated by Lasserus et al. in the case of Ag-Au nanoparticles [136]. Ag and Au are miscible and would normally be expected to form alloy nanoparticles, as observed in the majority of cases. However, by using sequential pick-up they, Lasserus et al. were able to produce core–shell nanoparticles of Ag@Au and even Au@Ag, in which the high surface energy material is in the shell. These remained stable after deposition onto a room temperature substrate, but heating to 573 K produced the alloy, as shown by the HAADF images of a single Ag@Au nanoparticle shown in Figure 16.

### 4.5. Other Structures

**Figure 17:**
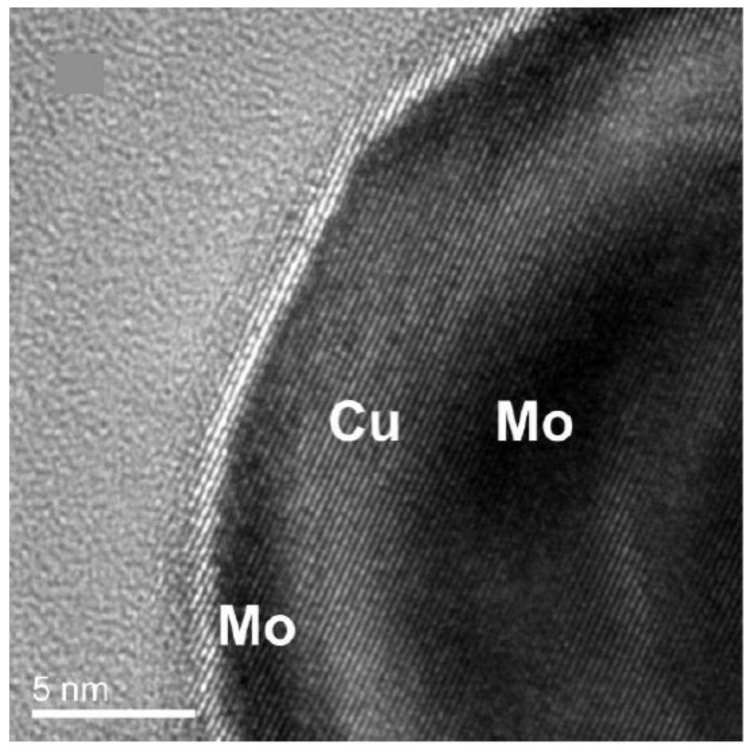
HRTEM image of a Mo@Cu@Mo nanoparticle produced by a segmented MoCu target in a sputter source. Reproduced from supplementary information to [23].

The discussion so far has been framed in the context of the three types of structure illustrated in Figure 1, but a number of other arrangements have been observed. Some of these have already been mentioned, including the core–shell structure with an asymmetric core, which evolves to the full Janus structure. There have also been several reports of two-element nanoparticles forming multiple shells, often described as “nano-onions”. For example, Figure 17 shows an HRTEM image of a Mo@Cu@Mo nanoparticle produced by a segmented MoCu target in a sputter source [23]. Two-element onion structures have also been observed in Co@Au@Co nanoparticles [86] and Ag@Cu@Ag nanoparticles [43]. In three-element synthesis, nano-onions become possible with different elements in each layer, for example, the Co@Ag@Au nanoparticle shown in Figure 5.

Another issue that has been overlooked in the discussion is graded alloys. All interfaces have been assumed to be sharp in the discussion thus far, but in many multi-element nanoparticle structures, there exist continuously graded alloys without abrupt boundaries. An example is Pd-Pt-Au nanoparticles, which consist of a graded alloy with a AuPt-rich core and a Pd-rich shell [41].

## 5. Technological Applications

It is evident that even with two elements, an impressive range of structures can be formed using gas-phase synthesis, including alloy, core–shell, Janus, nano-onion and graded alloy nanoparticles. The flexibility increases further when more elements are included in the synthesis, and the huge parameter space of three and more element materials has hardly been touched. Gas-phase synthesis also comes with excellent size and composition control, exercised in real time by simple adjustments to the synthesis conditions, and a free choice of elements. Thus, in terms of prototyping nanoparticles for optimized performance in any given application, the method is supreme. The main disadvantages are the relatively high cost and the low rate of production of the nanomaterials. At the very least, it is an efficient and rapid way to find the best structure, size and composition of nanoparticle for a given application, which can then be the goal for a mass manufacturing method. However, for some applications, especially in medicine, where the amount of material needed is small (typically 10 mg for a single patient dose) and has very high added value, the flux of gas-phase sources has improved [3] to the point where they can also be considered for manufacture. For the vast majority of medical applications, nanomaterials must be produced as a hydrosol, but there are now several ways to do this from a high- or ultra-high vacuum source. These include depositing the particles in vacuum onto a water-soluble layer such as PVA or PEG on a substrate in vacuum [153] or co-depositing the nanoparticles with water vapor onto a liquid-nitrogen-cooled substrate in vacuum [35]. We focus, therefore, on medical applications.

Magnetic nanoparticles have many important roles in medicine, including as MRI contrast enhancers, tracer particles in magnetic particle imaging (MPI) and heating elements in magnetic nanoparticle hyperthermia (MNH) treatment of cancer [1]. In all these applications, the materials currently used are Fe oxide (Fe_2_O_3_ or Fe_3_O_4_) particles, but it is well known that massive improvements in performance would become possible if the magnetic moment of pure Fe were available [154]. This is possible with the synthesis of core–shell nanoparticles with an Fe core and a noble metal shell that protects the core from oxidation and provides a more biocompatible surface that is easy to functionalize with ligands and possible targeting vectors. For example, Fe@Cu and Fe@Au both form stable core–shell nanoparticles. FeCo@Ag and FeCo@Au have also been produced by three-element sources [142,155], yielding an even higher magnetic moment in the core.

**Figure 18:**
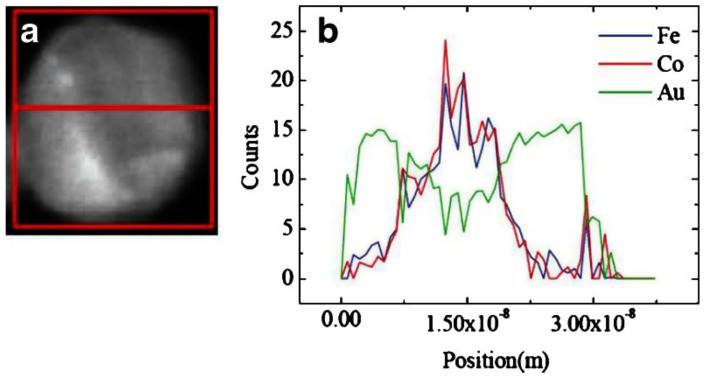
(**a**) HAADF image of a (FeCo)@Au nanoparticle used in an MRI study. (**b**) EDS line scan along the red line in a demonstrating the FeCo core and Au shell. Reproduced from [155].

Patra et al. produced (Fe_70_Co_30_)_50_Au_50_ core–shell nanoparticles with diameters around 25 nm in a sputter source with a composite target, and a typical example is shown in the HAADF image in Figure 18a [155]. The EDS line scan along a diameter (Figure 18b) clearly shows the FeCo core and Au shell. The particles were collected in a hydrosol by depositing them onto polyethylene-glycol- (PEG-) coated substrates to render them water-soluble. The particles were then incubated with human umbilical vein endothelial cells (HUVECs) and tested in vitro for MRI R2 signals. Their uptake in cells produced a strong R2 MRI contrast in comparison to cells without the nanoparticles. The nanoparticles were also found to inhibit the function of a pro-angiogenic growth factor (VEGF165) and thus would have some therapeutic effect against cancers. This combination of therapy and diagnosis labelled “theragnostics” is an area where multi-element nanoparticles can be extremely effective.

Magnetic nanoparticles are particularly effective as theragnostic agents, as they can produce diagnosis by MRI or MPI and treat cancers by MNH. However, other theragnostic combinations are possible, such as optical hyperthermia and diagnosis by X-rays. Optical hyperthermia of tumors exploits a near-infrared window at which tissue is relatively transparent, and the heating is provided by absorption by the surface plasmon resonance (SPR) in Au nanoshells or nanorods [1]. These structures are required to shift the SPR, which in a pure Au nanoparticle is in the visible part of the spectrum, to the near infrared. A much more effective alternative is to use nano-onions consisting of a metal shell, an intermediate oxide shell and a gold outer shell [156]. The Au in the shell also acts as a powerful absorber of X-rays, providing the diagnostic part of the theragnostic couple.

**Figure 19:**
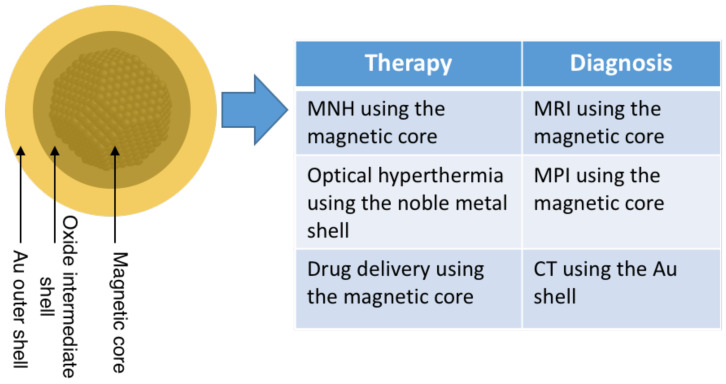
Core–shell–shell nanoparticle able to provide several therapeutic and diagnostic techniques.

Given the flexibility of gas-phase synthesis with the possibility to combine several elements, it is possible to design a nanoparticle, as illustrated in Figure 19, enabling several diagnostic and therapeutic modes. It would have a magnetic core of metallic Fe and an intermediate oxide shell to shift the SPR of the Au outer shell into the near infrared. For therapy, it could thus be used for magnetic hyperthermia, optical hyperthermia or drug delivery using external magnetic fields. For diagnosis, it could be used as an MRI contrast enhancer or an MPI tracer or provide sensitive detection by CT scans.

## 6. Conclusions

We reviewed the current literature on gas-phase synthesis of multi-element nanoparticles. The field’s 22-year history has demonstrated a rich variation of structures that include alloy, core–shell, Janus, nano-onion and graded alloy nanoparticles, in some cases with more than two elements. We showed that in most cases, the equilibrium structures can be understood by considering the balance of surface energies and enthalpy of mixing, considering the size dependence of both. It is also possible to produce out-of-equilibrium structures that are stable at room temperature. The huge flexibility and control over the nanoparticle structures allows for rapid optimization of performance in specific technical applications.

## Figures and Tables

**Table 1:** List of two-element nanoparticles reported.

Element 1	Element 2	Size	Surface Energy Difference (Jm^−2^)	Enthalpy of Mixing (kJ/mol)	Structure of Nanoparticle	Main Reference
		(nm)				
Mg	Ti	~20	−1.41	+20 [46]	Ti@Mg ^1^	[47]
	Ti	~10	−1.41	+20 [46]	Ti@Mg ^2^	[48]
	Ni	~20	−1.45	−12 [49]	Ni@Mg ^1^	[48]
	Cu	~20	−0.83	−9.8 [50]	Cu@Mg ^1^	[48]
	Pd	~5	−0.86	−43 [51]	Pd@Mg	[36]
Al	Fe	10	−1.73	−25 [52]	Fe@Al	[53]
	Yb	5–10	−0.34	−30 [54]	Al@Yb	[55]
	Au	1–2	—	—	Al dopant	[30,31]
Si	Co	~30	−1.01	NA (-)	Co@Si	[56]
	Co	~18	−1.01	NA (-)	alloy	[57]
	Mn	10–20	|2.16|	−35 [58]	alloy	[59]
	Ag	4–15	|0.56|	+2.5 [60]	C–S ^3^, Janus	[32]
Sc	Au	1–2	—	—	Sc dopant	[61]
Ti	V	5	|0.40|	NA (-)	alloy	[62]
	Ni	~20	−0.04	−33 [63]	Ni@Ti	[64]
	Ni	15–20	−0.04	−33 [63]	Ni@Ti	[65]
	Cu	8	|0.58|	−12 [66]	alloy	[67]
	Pt	1–2	|0.40|	−91 [68]	alloy^4^	[69]
	Pt	8	|0.40|	−91 [68]	alloy	[62]
	Au	1–2	—	NA (-)	Ti dopant	[61]
V	Pt	7	−0.87	−27 [63]	V@Pt	[62]
	Au	1–2	—	—	V dopant	[61]
Cr	Fe	2.8	1.2	+6.3 [70]	Fe@Cr	[34]
	Fe	10–50	1.2	+6.3 [70]	Fe@Cr	[71]
	Co	5–10	|1.01|	+2.5 [72]	alloy	[73]
	Co	6–7	1.01	+2.5 [72]	Co@Cr	[74]
	Ni	5	|1.31|	+6.4 [70]	alloy	[75]
	Ni	10–12	|1.31|	+6.4 [70]	alloy	[76]
	Au	1–2	—	—	Cr dopant	[61]
Mn	Fe	10–50	|0.96|	−4.5 [70]	alloy, Janus	[71]
	Au	1–2	—	—	Mn dopant	[61]
	Bi	~10	−3.16	NA (-)	Bi@Mn or Mn@Bi@Mn	[77]
Fe	Co	~12	|0.19|	−10.5 [78]	alloy	[79]
	Cu	2–3	−1.11	+11 [80]	Fe@Cu	[81]
	Cu	~20	|1.11|	+11 [80]	alloy	[82]
	Zn	~5	|2.09|	−2.2 [83]	alloy	[84]
	Zn	~20	−2.09	−2.2 [83]	Fe@Zn	[85]
	Ag	~15	|1.74|	NA (+)	Janus	[86]
	Ag	~20	|1.74|	NA (+)	Janus	[87]
	Ag	~20	|1.74|	NA (+)	Janus	[88]
	W	5–15	−0.81	+0.4 [70]	W@Fe	[89]
	Pt	4–8	|0.93|	−25 [70]	alloy	[90]
	Pt	8	|0.93|	−25 [70]	alloy	[91]
	Au	1	—	—	Fe dopant	[31]
	Au	1–2	—	—	Fe dopant	[61]
	Au	2–3	−1.78	+10 [92]	Fe@Au	[81]
	Au	~10	−1.78	+10 [92]	Fe@Au	[82]
	Au	10	−1.78	+10 [92]	Fe@Au ^5^	[93]
Co	Ag	2–5	−1.55	NA (+)	Co@Ag	[94]
	Ag	2–4	|1.55|	NA (+)	alloy	[95]
	Ag	7–27	−1.55	NA (+)	Co@Ag	[89]
	Mo	2–10	−0.57	NA (-)	Mo@Co	[89]
	Sm	5	|1.46|	−99 [96]	alloy	[37]
	Sm	7	|1.46|	−99 [96]	alloy	[97]
	Sm	~40	|1.46|	−99 [96]	alloy	[98]
	Pt	2–4	0.74	−109 [99]	alloy	[95]
	Pt	2–6	0.74	−109 [99]	alloy	[100]
	Au	1	—	—	Co dopant	[31]
	Au	1–2	—	—	Co dopant	[61]
	Au	10	−1.59	+7 [101]	Co@Au	[102]
	Au	5–10	−1.59	+7 [101]	Co@Au	[103]
	Au	8	−1.59	+7 [101]	Co@Au	[104]
	Au	7	−1.59	+7 [101]	Co@Au	[105]
	Au	5–15	−1.59	+7 [101]	Co@Au	[106]
	Au	12	−1.59	+7 [101]	Co@Au	[86]
	Au	5	|1.59|	+7 [101]	Janus	[107]
Ni	Cu	20–50	−0.62	+3.7 [80]	Ni@Cu	[108]
	Cu	~30	|0.62|	+3.7 [80]	alloy	[109]
	Mo	4	|0.87|	NA (-)	alloy	[110]
	Ag	2–5	−1.25	NA (+)	Ni@Ag	[94]
	Pt	1–2	|0.44|	−9.5 [111]	alloy	[112]
	Au	1	—	—	Ni dopant	[31]
	Au	1–2	—	—	Ni dopant	[61]
	Au	2–5	−1.29	+3 [92]	Ni@Au	[94]
Cu	Mo	10–60	|1.49|	NA (+)	alloy, C–S, Janus	[23]
	Pd	2–5	|0.03|	−45 [113]	alloy	[114]
	Ag	5–8	|0.63|	+3.5 [115]	alloy	[116]
	Ag	12	−0.63	+3.5 [115]	Cu@Ag, Janus	[44]
	Ag	5–15	−0.63	+3.5 [115]	Cu@Ag ^6^	[43]
	Ag	10–20	|0.63|	+3.5 [115]	Janus	[117]
	W	5–8	|1.92|	NA (+)	alloy	[116]
	Au	1–2	—	−29 [115]	Cu dopant	[30]
	Au	~2	|0.67|	−29 [115]	alloy	[118]
	Au	~4	|0.67|	−29 [115]	alloy	[119,120]
	Au	<5	|0.67|	−29 [115]	alloy	[121]
Y	Co	<10	|1.34|	NA (-)	alloy	[122]
	Co	8–10	|1.34|	NA (-)	alloy	[123]
	Pt	4–10	|0.60|	−104 [63]	alloy ^7^	[124]
	Au	1–2	—	−79 [63]	Y dopant	[30]
Ru	Pt	~5	|1.28|	—	alloy	[125]
Pd	Ag	5–10	|0.66|	−5 [126]	alloy	[73]
	Pt	<6	0.15	−4.3 [70]	Pd@Pt	[127]
	Pt	11	|0.15|	−4.3 [70]	alloy	[128]
	Pt	<5	|0.15|	−4.3 [70]	core–shell ^8^	[129]
	Au	8–22	|0.70|	−8.4 [130]	alloy	[131]
	Au	1–5	|0.70|	−8.4 [130]	alloy	[132]
	Au	5–10	|0.70|	−8.4 [130]	alloy	[73]
	Au	5	|0.70|	−8.4 [130]	alloy	[133]
	Au	3	|0.70|	−8.4 [130]	alloy	[134]
Ag	Au	8–22	|0.04|	−17 [115]	alloy	[131]
	Au	<5	|0.04|	−17 [115]	alloy	[33]
	Au	5–10	−0.04	−17 [115]	Ag@Au	[103]
	Au	2–5	−0.04	−17 [115]	Ag@Au	[39]
	Au	8–10	|0.04|	−17 [115]	alloy	[135]
	Au	3–4	|0.04|	−17 [115]	C–S, alloy ^9^	[136]
	Au	5–20	|0.04|	−17 [115]	alloy	[137]
In	Au	1–2	—	—	In dopant	[30]
Pt	Au	5–8	|0.85|	NA (+)	alloy	[116]
	Au	1.5–3.5	|0.85|	NA (+)	alloy	[138]
	Au	2–3	|0.85|	NA (+)	alloy	[139]

^1^ Alloy nanoparticles formed after hydrogenation of Mg; ^2^ before hydrogenation of Mg; ^3^ C–S stands for core–satellite structure, that is, multiple cores with a common shell; ^4^ becomes Pt@Ti on oxidation; ^5^ also observed the formation of a Au shell beneath the Fe surface; ^6^ observation of patterned shell (“Ukidama” nanoparticles) and Ag@Cu@Ag structure; ^7^ a Pt-rich shell forms on oxidation; ^8^ PtPd@Pt for Pt-rich and PtPd@Pd for Pd-rich; ^9^ both Ag@Au and Au@Ag NPs formed in liquid He droplets, and in both cases they alloy with annealing.

**Table 2:** List of three-element nanoparticles reported.

Elements	Structure	Size (nm)	Reference
Fe, Co, Ag	(FeCo)@Ag	~20	[142]
Fe, Co, Au	(FeCo)@Au	~14	[142]
Pd, Ag, Au	Ternary alloy	~5	[33]
Co, Ag, Au	Co@Ag@Au	10	[103]
Si, Fe, Ag	FeAg@Si ^1^	10–50	[143,144]
Pd, Pt, Au	(AuPt)@Pd ^2^	10	[41]

^1^ The FeAg core in these nanoparticles is a Janus particle; ^2^ the nanoparticles consist of a graded alloy with a AuPt-rich core and a Pd-rich shell.

**Table 3:** Surface energies of the elements listed in Table 1.

Element	Surface Energy *Crystalium* (Jm^−2^)	Surface Energy Liquid Metal (Jm^−2^)
Mg	0.59	0.57
Al	0.80	0.87
Si	1.33	0.80
Sc	1.25	0.87
Ti	2.00	1.50
V	2.47	1.90
Cr	3.35	1.69
Mn	3.49	1.10
Fe	2.53	1.83
Co	2.34	1.83
Ni	2.04	1.74
Cu	1.42	1.31
Zn	0.44	0.77
Y	1.00	—
Mo	2.91	2.13
Ru	2.88	2.22
Pd	1.45	1.48
Ag	0.79	0.91
In	0.31	0.56
Sm	0.88	—
Yb	0.46	—
W	3.34	2.34
Pt	1.60	1.86
Au	0.75	1.13
Bi	0.24	0.38

## Appendix A

**Table A1:** Summary of two-element nanoparticles reported since 1999 in date order and including method of manufacture.

Elements	Synthesis Method	Particle Size	Internal Structure	Comment	Reference	Elements	Synthesis Method	Particle Size	Internal Structure	Comment	Reference
AuCu, AuAl, AuY, AuIn	Dual laser ablation	100 atoms	Dopants in small Au clusters	Au clusters doped with 1 or 2 atoms of other metals to study magic numbers (1999)	[30]	AuAl, AuFe, AuCo, AuNi	Dual laser ablation	~10 atoms	Small Au_n_X_m_ clusters	Studied effect of time delay between laser pulses (2000)	[31]
AuCu	Laser ablation from alloy	~2 nm	Alloy	Structural study. Atomic structure differed from bulk alloy (2001)	[118]	SmCo	Laser ablation from alloy	~5 nm	Alloy	(2001)	[37]
AuSc, AuTi, AuV, AuCr, AuMn, AuFe, AuCo, AuNi	Dual laser ablation	<50 atoms	Dopants in small Au clusters	Study of magic numbers in Au clusters doped with transition metal atoms (2003)	[61]	NiAu, NiAg, CoAg	Laser ablation of alloy target	2–5 nm	Ni@Au, Ni@Ag, Co@Ag	Size evolution of optical properties (2003)	[94]
SmCo	Sputter source with alloy target	7 nm	Alloy	Magnetic properties of NPs before and after “in-flight” annealing (2003)	[97]	FePt	Sputter source with alloy target	4–8 nm	Alloy	Phase transformation from the disordered fcc phase to ordered fct phase in FePt nanoparticles by “in-flight” annealing (2003)	[90]
FeCo	Hollow cathode arc source with alloy cathode	~12 nm	Alloy	Formation of high-moment FeCo alloy nanoparticles (2004)	[79]	CoAg CoPt	Dual laser ablation	2–4 nm	Alloy	Study of changes in magnetic anisotropy (2004)	[95]
CoSi	Sputter source with multiple targets	~30 nm	Core–shell with Si rich shells	Magnetic properties of Co@Si clusters compared with Co@CoO (2005)	[56]	AuAg, AuPd	Laser ablation of alloy pellet	8–22 nm	Alloy	Study of plasmon resonances (2006)	[131]
FePt	Sputter source with alloy target	8 nm	Alloy	Obtention of ordered tetragonal phase (2006)	[91]	CoAu	Sputter source with alloy target	5–15 nm	Co@Au	Magnetic behavior of Co@Au (2007)	[106]
AuPd	Sputter source with alloy target	1–5 nm	Alloy	Icosahedral particles (2008)	[132]	CoAu, FeAg	Sputter source with alloy target	12 nm (Au), ~15 nm (FeAg)	CoAu, Janus (FeAg)	Transition between core–shell and Janus structures (2008)	[86]
CrCo, AuPd, AgPd	Spark ablation source with alloy electrodes (CrCo) or different elemental electrodes (AuPd, AgPd)	5–10 nm	Alloy	Comparison of elemental mixing using alloy or separate elemental electrodes (2008)	[73]	FeCu, FeAu	Thermal core plus thermal shell	~2–3 nm	Core–shell	Modification of core atomic structure by changing the shell thickness (2010)	[81]
AgCu, CuW, PtAu	Spark ablation source with sintered electrodes (AgCu, CuW) or two different electrodes (PtAu)	~5–8 nm	Alloy	Nanocrystalline phases found for AgCu and PtAu (2010)	[116]	AuPd	Sputter source with alloy target	5 nm	Alloy	Study of the effect of temperature on structure (2010)	[133]
AuCo	Sputter source with sectioned target	5 nm	Janus	HRTEM showed details of the Co/Au interface (2010)	[107]	YCo	Sputter source with sectioned target	<10 nm	Alloy	Study of magnetic behavior for different stoichiometries (2011)	[122]
AgFe	Sputter source with sectioned target	~20 nm	Janus	In-flight thermal annealing performed (2011)	[88]	CoPt	Laser ablation of alloy target	2–6 nm	Alloy	Morphology study of CoPt clusters (2011)	[100]
YCo	Sputter source with composite target	8–10 nm	Alloy	Magnetic study of NPs with different crystal structures (2011)	[123]	AuCu	Sputter source with alloy target	~4 nm	Graded alloy with either Cu-rich core or Au-rich core	(2011), instability of Au@Cu core–shell nanoparticles (Au diffused to the shell) (2012)	[119,120]
AgAu	Sputter sources with independent targets	<5 nm	Alloy	Fine tuning of the composition achieved via the multiple target cluster source (2012)	[33]	CuAg	Dual laser ablation	12 nm	Co@Ag, Janus	Description of the transition from core–shell to Janus structures (2012)	[44]
FeCr	Thermal core plus thermal shell	2.8 nm	Fe@Cr	Controlled shell thickness to observe onset of exchange bias (2013)	[34]	SmCo	Sputter source with sectioned target	~40 nm	Alloy	Study of the growing mechanism and coercivity with sputter current (2013)	[98]
MoCu	Sputter source with segmented target	10–60 nm	Alloy, core–shell, Janus	Structural motifs controlled by changing the source parameters (2013)	[23]	CoAu	Sputter source with alloy target	10 nm	Alloy or Co@Au depending on deposition temperature	Magnetic and structural behavior of Co@Au with deposition temperature (2013)	[102]
MgNi, MgCu, MgTi	Sputter source with segmented target	10–20 nm	Ni@Mg, Cu@Mg, Ti@Mg	Phase separation seen in the case of MgNi and MgCu to produce core–shell structure after hydrogenation (2014)	[48]	AuAg, AuCo	Sputter sources with 3 targets	5–10 nm	Co@Au, Ag@Au	Changing the position of the magnetrons within the aggregation source enabled swapping core and shell materials (2014)	[103]
NiCu	Sputter source plus sputter coater	20–50 nm	Ni@Cu	Shell thickness up to 5 nm (2014)	[108]	AlYb	Sputter source with sectioned target	5–10 nm	Al@Yb	Oxidation experiments on the Yb shell (2014)	[55]
PtY	Sputter source with alloy target	4–10 nm	Alloy in as-prepared samples, thin Pt-rich shell after oxidation	Study of PtY NPs as catalysts for the oxygen reduction reaction (2014)	[124]	AgSi	Sputter source with multiple targets	4–15 nm	Core-satellite, Janus	Study of morphology by variation of operational parameters and MD study (2014)	[32]
FeAl	Sputter source with multiple targets	10 nm	Fe@Al	Oxidation of the shell produces an alumina shell (2014)	[53]	AgCu	Liquid He droplet source	2–5 nm	Ag@Au	Multiple cores observed above a critical size (2015)	[39]
PdMg	Sputter source with segmented target	~5 nm	Pd@Mg	Synthesis of catalytic particles (2015)	[36]	AuCo	Sputter source with multiple targets	8 nm	Co@Au	Icosahedral core–shell Co@Au nanoparticles and novel structure (icosahedral Co core surrounded by fcc Au facets) (2015)	[104]
NiCr	Sputter source with alloy target	5 nm	Alloy	Deterioration of magnetic properties due to Cr segregation observed (2015)	[75]	TiV, PtV, PtTi	Sputter source with multiple targets	5–8 nm	TiV alloy, V@Pt, TiPt alloy	Structure tunable with source parameters (2015)	[62]
RuPt	Sputter source with multiple targets	~5 nm	Alloy	Electrochemical performance of RuPt alloy nanoparticles (2015)	[125]	CoAg, FeW, MoCo	Sputter source with composite target	7–27 nm (CoAg), 5–15 nm (FeW), 2–10 nm (MoCo)	Co@Ag, W@Fe, Mo@Co	Minimum size for spontaneous core–shell formation (2015)	[89]
AgCu	Sputter source with multiple targets	5–15 nm	Cu@Ag	Cu@Ag structure in Cu-rich particles and Ag@Cu@Ag in Ag-rich particles. “Ukidama” nanoparticles observed (2016)	[43]	FeCu	Sputter source plus thermal shell	~20 nm	Alloy	(2016)	[82]
FeAg	Sputter source plus thermal shell	~20 nm	Core with islanded shell (Janus)	(2016)	[87]	FeAu	Sputter source plus thermal shell	~10 nm	Core–shell	(2016)	[82]
PtNi	Sputter source with alloy target	1–2 nm	Alloy	High catalytic activity for methanol electro-oxidation when decorated on carbon nanotubes (2016)	[112]	NiCr	Sputter source with alloy target	10–12 nm	Alloy	Study of Cr segregation and its effect on magnetic properties (2016)	[76]
MnBi	Sputter source with alloy target	~10 nm	Bi@Mn or Mn@Bi@Mn	HRTEM study showing crystalline Bi and amorphous Mn (2016)	[77]	CoSi	Sputter source with composite target	~18 nm	Alloy	Study of the coercivity of the NPs (2016)	[57]
MgTi	Sputter source with segmented target	~20 nm	Ti@Mg	Showed nucleation induced by the introduction of trace gases (2017)	[47]	PdPt	Dual laser ablation	<6 nm	Pd@Pt	Study of hydrogen detection when deposited on ZnO nanorods (2017)	[127]
AgAu	Sputter source with sectioned target	8–10 nm	Alloy	Characterization of AgAu in a SiO_2_ matrix (2017)	[135]	NiTi	Sputter source plus sputter coater	~20 nm	Core–shell (Ni@Ti)	Demonstrated independent control of shell thickness (2017)	[64]
AgAu	Liquid He droplet source	2 nm	Au@Ag or Ag@Au (depending on order of pick-up cells) transforming to alloy after annealing	HR TEM study of the alloying of metastable core–shell nanoparticles with annealing (2018)	[136]	AuCu	Sputter source with multiple targets	<5 nm	Alloy	Nanoparticles were deposited in MgO as catalysts (2018)	[121]
TiCu	Sputter source with alloy target	8 nm	Alloy	Growth mechanism modelled by a nucleation model (2018)	[67]	CoAu	Sputter source with alloy target	7 nm	Co@Au	Trimodal distribution and different structures when using pulsed sputtering instead of DC (2018)	[105]
MnSi	Sputter source with composite target	10–20 nm	Alloy	Study of skyrmionic properties of NPs (2018)	[59]	NiMo	Sputter source with alloy target	4 nm	Alloy	Production of NiMoS with a reactive atmosphere of H_2_S (2018)	[110]
PtTi	Sputter source with alloy target	1–2 nm	Alloy, but becomes core–shell (Pt@Ti) with oxidation	Multicore Pt atoms observed after ambient exposure in the largest clusters (2018)	[69]	NiCu	Sputter source plus sputter coater	~30 nm	Alloy	Enriched Cu shell observed for high Cu content (2019)	[109]
PdPt	Laser ablation of a sectioned plate	11 nm	Alloy	Formation of nanoparticle graphene composites (2019)	[128]	AuPd	Sputter source plus sputter coater	3 nm	Alloy	Even distribution of elements confirmed by EDS (2019)	[134]
FeAu	Sputter source with multiple targets	10 nm	Core–shell (Fe@Au) and solid solution, where Au shell forms within the Fe core	FeAu nanocubes where Au occupied specific sites. Atomistic simulation of the growth mechanism (2019)	[93]	FeCr, FeMn	Spark ablation source with alloy electrodes plus thermal treatment	10–50 nm	Fe@Cr, alloy, Janus (FeMn)	Incorporation of H_2_ as carrier gas led to core–shell morphologies, and subsequent annealing led to Janus FeMn nanoparticles (2019)	[71]
PdPt	Sputter source with multiple targets	<5 nm	Core–shell PtPd@Pt for Pt-rich and PtPd@Pd for Pd-rich	Out-of-equilibrium structures due to kinetic trapping. Verified by MD simulations (2020)	[129]	PdCu	Sputter source with multiple targets	2–5 nm	alloy	Alloy nanoparticles deposited into liquid polymer (2020)	[114]
PtAu	Sputter source with multiple targets	1.5–3.5 nm	Alloy	Deposition into liquid PEG. High Pt content prevented agglomeration (2020)	[138]	AuPt	Dual laser ablation	2–3 nm	Alloy	UV sensitivity increased in ZnO decorated with nanoparticles (2020)	[139]
AgCu	Sputter source plus sputter coater	10–20 nm	Janus	Coating with a tubular magnetron in which magnetic trapping increased thickness of the shell (2020)	[117]	CoCr	Sputter source with alloy target	6–7 nm	alloy, Co@Cr,	Minimum size of nanoparticle for the spontaneous formation of Co@Cr (2020)	[74]
NiTi	Sputter source plus sputter coater	15–20 nm	(Ni@Ti)	Ni@Ti NPs with thicker shells due to an arrow-shaped configuration of the aggregation chamber (2020)	[65]	AgAu	Sputter source with composite target	5–20 nm	Alloy	Study of the effect of re-deposition in the composite target (2020)	[137]
ZnFe	Sputter source plus sputter coater	~5 nm	Alloy	Particles formed a galvanic couple, promoting faster oxidation of Zn (2021)	[84]	ZnFe	Sputter source with multiple targets	~20 nm	Fe@Zn/ZnO (Flower-like nanoparticles)	MD simulations used to understand particle growth (2021)	[85]
